# Temperature-Related Corrosion Resistance of AISI 1010 Carbon Steel in Sulfolane

**DOI:** 10.3390/ma13112563

**Published:** 2020-06-04

**Authors:** Julian Kubisztal, Bożena Łosiewicz, Paulina Dybal, Violetta Kozik, Andrzej Bak

**Affiliations:** 1 Institute of Materials Engineering, University of Silesia in Katowice, 75 Pulku Piechoty 1A, 41-500 Chorzow, Poland; bozena.losiewicz@us.edu.pl; 2 Institute of Chemistry, University of Silesia in Katowice, Szkolna 9, 40-007 Katowice, Poland; pdybal@us.edu.pl (P.D.); violetta.kozik@us.edu.pl (V.K.)

**Keywords:** AISI 1010 carbon steel, sulfolane, corrosion resistance, solvent temperature, electrochemical techniques

## Abstract

Sulfolane-induced corrosion can lead to severe impairment in industrial systems. Therefore, determination of solvent corrosivity is valid. Under standard conditions, pure sulfolane is considered to be thermally stable and chemically inert, hence non-aggressive towards carbon/stainless steel. Unfortunately, the sulfolane-evoked corrosion of the industrial installations is observed for sulfolane-based systems polluted by small quantities of oxygen, water and some oxidizing agents. Moreover, sulfolane decomposition with formation of corrosive (by-)products can be escalated by some process parameters, e.g., temperature. The main objective of this study was to determine the corrosion resistance of AISI 1010 steel immersed in sulfolane at temperatures ranging from 25 to 230 °C. Evaluation of the corrosion damage was carried out using electrochemical techniques and scanning probe/electron microscopy, respectively. The general corrosion tendency, corrosion rate and surface corrosion degree were taken into account as well. It was noticed that the corrosion rate linearly increases with the enhancement of sulfolane temperature. Moreover, the interfacial reaction of steel with sulfolane resulted in the formation of corrosion product layer, which is a physical barrier between the corrosive environment and steel improving corrosion resistance of the latter. In fact, the increment of the sulfolane temperature caused a gradual breakdown of the protective layer and the increase in the corrosion degree of the investigated steel. Finally, it was found that the corrosion degree doubles approximately every 42 °C.

## 1. Introduction

A worldwide rise of atmospheric pollution due to the expansion of industrial/agricultural areas and urban settlements is a peculiar ‘landmark’ of the modern civilization. The global ecosystem is being confronted with volatile organic compounds (VOCs) as well as inorganic odorous compounds (VICs) that pose hazards to the health of human beings and plants vegetation forming a significant part of indoors/outdoors pollution [1,2,3]. The individual sulfur derivatives exert some irritant and toxic effects, being classified as a potential mutagenic and carcinogenic risk factors [4]. In particular, a valid subset of air/water/soil pollutants, composed of sulfur-containing derivatives and their metabolites, namely ‘old devils of green chemistry’, has detrimental impact on human health following long-term exposure [5]. The demand for environmental protection and necessity to diminish the ecosystem burden is the major driving force that pushes the industry decision-makers to develop and/or optimize the sustainable, operationally simple manufacturing technologies; however, some industrially-engineered solvents are still harmful to nature [6]. The reduction of sulfur-based compounds (RSCs) from the unprocessed natural/industrial off-gasses can be achieved in a liquid/liquid or liquid/vapor extraction processes, although the design of the ‘green’ solvents and cost-effective, large-scale procedures represents a relevant challenge [7]. 

An appealing alternative to extensively employed extractive liquids in the dearomatization of petroleum fractions or sweetening of sour gases in the petrol-related industry is sulfolane (SFL) [8]. Although the spelling ‘sulfolane’ may be astonishing for British English users this name has been accepted as the generic name for hydrogenated sulfones of butadiene with a four-membered carbon ring and sulfonyl functional group (=SO_2_) [9]. On the other hand, sulfolane is also known under a variety of synonyms/numbers including thiolane 1,1-dioxane (IUPAC), 2,3,4,5-tetrahydrothiophene-1,1-dioxide (systematic), thiocyclopentane-1,1-dioxide, (cyclo) tetramethylene sulphone, dihydrobutadiene sulphone, sulphoxaline, 126-33-0 (CAS) or 204-783-1 (EINECS), respectively. SFL (C_4_H_8_O_2_S) is a versatile dipolar aprotic solvent with a high oxidation potential (> 6.35 V) that is highly soluble in water (1.266 × 10^6^ mg/L at 20 °C) [10,11]. Unfortunately, the extensive SFL production and usage in industry has brought an increase in issues with liquid storage security; unintended leakage from extraction units in refineries or gas plants into the surrounding environment proved inevitable, severely affecting the ecology of neighboring areas [12]. Once in the environment, SFL is vulnerable to long-distance movement into groundwater and subsequent migration to domestic wells, deteriorating water sensory properties [13]. Due to restricted data pertaining to absorption, distribution, metabolism, excretion and toxicity (ADME-Tox) related to acute inhalation or intraperitoneal exposure to SFL-rich aqueous solutions, the sulfolane-based liquids in the environmental/biological systems were targeted for extensive scrutiny [14]. Potential neurotoxicity of dose-related SFL/water mixtures was investigated in the inhalation studies of aerosolized liquid in various rodent species [15]. The fate of well-absorbed radio-labeled [2,5-^14^C] or [^3^H] or [^35^S] sulfolane was monitored to specify in vivo metabolic path(s) of unchanged sulfolane content or its metabolites following oral/intravenous/dermal administration. Since the toxicokinetic pattern of SFL disposition and clearance cannot be directly foreseen in the human body based on the quantitative ADME-Tox rodent models, it is therefore still not clear whether SFL can be regarded as ‘magic extractor or bad actor’ [16,17]. In this context, it is natural to raise a question about environmentally friendly and low-cost chemical and/or biological protocols for treatment of SFL contaminated groundwater or soil wash water. The operational parameters for bioremediation potential of indigenous microbes at the polluted site were scrutinized revealing that the efficiency of SFL biodegradation is strongly dependent on the dissolved oxygen (DO) level according to the aerobic mineralization formula [18]:

(1) $$C_{4}H_{8}O_{2}S+6.5O_{2}\to 4{\mathrm{CO}}_{2}+3H_{2}O+2H^{+}+{\mathrm{SO}}_{4}^{2-}$$

The application of a bio-augmentation strategy that assumes the employment of co-aggregated bacterial consortia considerably improved the SFL removal [19]. The nutritional supplement of diverse microbial communities should be controlled at the sufficient C:N:P ratio level to ensure the enhanced in situ degradation of the target pollutant. In other words, moderate temperature and proper addition of bio-stimulating agents that increase the concentration of the biomass in the system seem to refine SFL bioremediation capability. Ex situ soil washing conjugated with the advanced oxidation processes (AOPs) were applied for the purification of SFL-containing soil wash water using the strong oxidizing potential of hydroxyl radicals (·OH) [20]. A multitude of processes for ·OH radical generation makes the AOPs approach an attractive alternative to the conventional SFL/water treatment methods. The impact of experimental parameters including water/soil ratio, number of extraction cycles or shaking time was analyzed for the synthetic (model) water and the real SFL-contaminated groundwater samples [21]. The oxidation-related treatment of SFL-polluted soil wash water was performed using UV/H_2_O_2_, UV/O_3_, alkaline ozonation and neutral Fenton reagents coupled with pH, chemical dosage of H_2_O_2_ and ethylenediaminetetraacetic acid chelated iron (FeEDTA), O_3_ flow rate and ultraviolet-C light controlling, respectively. The removal efficiency (RE) of SFL pollutant (>99%) transferred (82–93%) to aqueous phase was found for the scrutinized water/soil fractions along with mixing periods employing UVC irradiation and adequate H_2_O_2_ concentration (200 mg/L in undiluted sample), whereas neutral Fenton reagents performed pretty poorly (RE < 50%) [22]. It seems that AOPs revealed noticeable potential to degrade SFL in the contaminated water with the generation of non-toxic (by-)products and comprehensive SFL mineralization [23].

Due to shortcomings and operational costs of SFL-polluted soil/water treatment a question naturally appears about the main causes for unforeseen/accidental SFL leakage, as it is known that pure liquid is non-aggressive to steel under the standard operating conditions [24]. Unfortunately, the SFL-induced corrosion of the industrial installations is observed for SFL-based systems polluted by small quantities of oxygen, water and oxidizing agents such as chlorates, nitrates or peroxides [25]. Moreover, the SFL decomposition with formation of corrosive (by-)products can be escalated by some process parameters, e.g., temperature [26]. The sulfolane synthesis, application of sulfolane as an extractive solvent due to its ‘unique’ physicochemical properties, the potential of sulfolane to cause equipment corrosion and subsequent spills, the possible risk for groundwater contamination, danger for human health and ways of sulfolane biodegradation are reviewed briefly in our previous works [16,25].

It should be emphasized that there is a gap on the path leading from data to knowledge pertaining to the detailed quantitative assessment of the effect exerted by individual impurities or operational variables on the SFL-induced corrosion of carbon and alloyed steels. In fact, carbon steel is quite commonly used in the oil and gas industry due to its availability, constructability and relative low cost of production. On the other hand, the susceptibility to corrosion (low corrosion resistance) imposes boundaries on the practical longevity of carbon steel applications, petrochemical pipes are especially prone to corrosion damage due to the erosive/corrosive nature of the produced fluids or gases [26]. Thus, the principal objective of the presented study was to analyze the impact of sulfolane temperature on general corrosion tendency, corrosion rate and corrosion degree of AISI 1010 steel. 

## 2. Materials and Methods 

### 2.1. Set for Corrosion of AISI 1010 Steel in Sulfolane

The test material was AISI 1010 low carbon steel in the annealed state containing 0.1 wt.% C. Before the corrosion test, the steel samples were degreased in acetone. The corrosion process of as-obtained AISI 1010 steel (89 × 20 × 2 mm^3^) in sulfolane (chemically pure ≥ 99%, ≤ 0.2 vol.% of water) was conducted in a home-made sealed vessel. The detailed description of the vessel construction and the equipment used in corrosion test can be found elsewhere [25]. The sulfolane temperature was set to 25, 95, 180 and 230 °C. For each temperature the immersion time was 96 h. To reduce contact of sulfolane with air, a protective layer of inert gas (99.995% Ar) was formed over its surface. After 96 h of experiment, the vessel was cooled and the AISI 1010 steel electrodes were disassembled and rinsed in acetone.

### 2.2. Electrochemical Tests of AISI 1010 Steel 

A series of electrochemical tests were carried out for AISI 1010 steel covered with corrosion products formed after 96 h of immersion in the sulfolane at temperatures of 25, 95, 180 and 230 °C. Measurements were conducted in a thermostated three-electrode cell filled with sulfolane at the temperature of 25 °C using electrochemical system PARSTAT 2273 (Princeton Applied Research, Oak Ridge, TN, USA). Working and counter electrodes were AISI 1010 steel and platinum plates with geometric surface areas of 8 and 100 cm^2^, respectively. The reference electrode was a saturated calomel electrode (SCE) placed ca. 0.2 cm from the working electrode using a Luggin capillary. Ohmic drop Δ*E* = *jR*_S_ (V) was estimated using measured current density (*j*) and solution resistance (*R*_s_). Solution resistance was calculated using equation *R*_s_ = *lκ*^−1^ (Ω·cm^2^) where *l* is the distance between Luggin capillary and AISI 1010 steel surface (0.2 cm), *κ* is the sulfolane conductivity (0.35 μS·cm^−1^ at 25 °C [27]). The sulfolane resistance was equal to 0.57 MΩ·cm^2^. The open circuit potential (*OCP*) was registered for 60 min. Next, polarization curves *j* = *f*(*E*) (*E* is the electrode potential) were recorded in the range ± 150 mV_SCE_ versus *OCP* using the linear sweep voltammetry technique with the potential sweep rate *v* = 10 mV·min^−1^. Using *j* = *f*(*E*) curves with compensated ohmic drop and equation:

(2) $$j=j_{\mathrm{corr}}\left\{e^{\left[\frac{\ln10\left(E-E_{\mathrm{corr}}\right)}{\beta_{a}}\right]}-e^{-\left[\frac{\ln10\left(E-E_{\mathrm{corr}}\right)}{\beta_{c}}\right]}\right\}$$

corrosion current density (*j*_corr_), corrosion potential (*E*_corr_) as well as anodic (*β*_a_) and cathodic (*β*_c_) Tafel slopes were determined. In addition, the Stern-Geary coefficient (*B*), polarization resistance (*R*_p_) and corrosion rate (*CR*) were calculated according to the following equations:

(3) $$B=\frac{\beta_{a}\beta_{c}}{\ln10\left(\beta_{a}+\beta_{c}\right)}$$

(4) $$R_{p}=\frac{B}{j_{\mathrm{corr}}}$$

(5) $$CR=k\frac{EW}{\rho }j_{\mathrm{corr}}$$

where *ρ* is the material density, *EW* is the material equivalent weight and *k* is a coefficient which determines the unit of corrosion rate. For the investigated AISI 1010 steel electrodes *ρ* = 7.86 g·cm^−3^, *EW* = 27.923 (valence for Fe was assumed as 2) and *k* = 3.27·10^−3^ mm·g·μA^−1^·cm^−1^·year^−1^, which gives the corrosion current density expressed in μA·cm^−2^ corrosion rate in mm·year^−1^. Note that low value of the sulfolane conductivity at 25 °C causes the measured current to be very small, i.e., in the of order of nanoamps or smaller, which may lead to some noise. Therefore, to visualize and interpret the obtained data more accurately, noise was removed using Savitzky-Golay smoothing algorithm [28] and OriginPro 2018 software (OriginLab, Northampton, MA, USA). 

### 2.3. Surface Analysis of AISI 1010 Steel 

Qualitative and quantitative surface analysis of AISI 1010 steel electrodes was carried out using scanning electron microscope (SEM) JEOL JSM-6480 (JEOL Ltd., Tokyo, Japan), equipped with an energy dispersive X-ray spectroscopy (EDS, JEOL Ltd., Tokyo, Japan). Obtained SEM images were converted to black and white (black color corresponds to the corroded areas) and next the corrosion degree (*CD*) was calculated according to the following equation:

(6) $$CD=\frac{\mathrm{total}\;\mathrm{corroded}\;\mathrm{area}}{\mathrm{total}\;\mathrm{area}\;\mathrm{of}\;\mathrm{an}\;\mathrm{image}}100\%$$

The EDS technique allowed to determine the distribution of iron and carbon elements on the surface of AISI 1010 steel. 

Contact potential difference (*CPD*) maps were registered for the studied electrodes using a scanning electrochemical workstation PAR M370 (Princeton Applied Research, Oak Ridge, USA) equipped with a tungsten Kelvin probe (KP, ø500 μm, Princeton Applied Research, Oak Ridge, USA). The scanning area was 4 × 4 mm^2^ and the distance between the sample and the probe was c.a. 90 μm. Approximation of the histograms of the *CPD* values using Gaussian function given by the equation:

(7) $$g\left(CPD\right)=\frac{1}{\sigma \sqrt{2\pi }}e^{-\frac{1}{2}{\left(\frac{CPD-CPD_{\mathrm{av}}}{\sigma }\right)}^{2}}$$

allowed to estimate the average value of contact potential difference (*CPD*_av_) and its standard deviation (*σ*) that describe quantitatively the material surface properties. The detailed description of the preparation of histograms can be found elsewhere [29,30].

## 3. Results and Discussion

### 3.1. Corrosion Resistance Analysis of AISI 1010 Steel 

**Figure 1:**
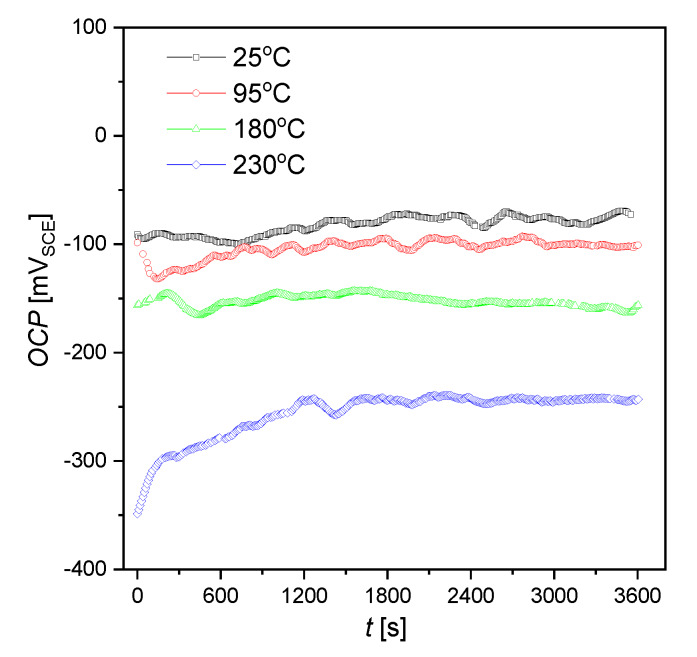
Open circuit potential for the AISI 1010 steel electrodes immersed in sulfolane at 25, 95, 180, and 230 °C; V_SCE_ is the electrode potential measured versus saturated calomel electrode.

Variations of the *OCP* measured for AISI 1010 steel electrodes in sulfolane for 60 min are illustrated in Figure 1. Using *OCP* = *f*(*t*) curves, the average values of *OCP* and corresponding standard errors (*SE*) were determined and are reported in Table 1. One can observe that the average value of *OCP* decreases with increased sulfolane temperature. In particular, when the temperature increases from 25 to 230 °C, the *OCP* value decreases by ca. 175 mV_SCE_ and the steel electrode reaches the most negative potential among all tested. Such behavior indicates an increase in the thermodynamic tendency to the corrosion process, thus deterioration of corrosion resistance of AISI 1010 steel. It was found that, up to about 180 °C, standard errors of the mean decrease and then increase, which may be caused by sulfolane decomposition at temperatures over 200 °C. On the one hand, sulfolane decomposition increases the corrosiveness of the environment. On the other hand, the heterogeneity of the surface geometry and/or chemical composition causes that some of its areas are more electrochemically active than others. These two factors generate some distribution of the potential on the material surface. It should be added that corrosion product layer influences the electrochemical parameters as well. Nevertheless, after 60 min relatively stable value of open circuit potential can be observed for all the investigated electrodes.

**Figure 2:**
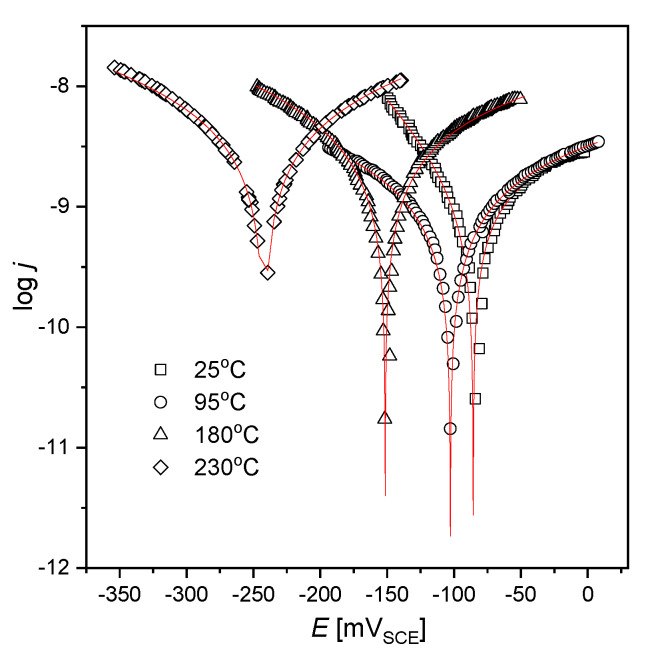
Polarization curves for the AISI 1010 steel electrodes immersed in sulfolane at 25, 95, 180, and 230 °C; symbols—experimental data, solid lines—fit of the Equation (2); V_SCE_ is the electrode potential measured versus saturated calomel electrode.

Polarization curves for the AISI 1010 steel electrodes immersed in sulfolane at 25, 95, 180, and 230 °C are shown in Figure 2, while electrochemical parameters obtained using registered curves and Equation (2) are reported in Table 2.

**Figure 3:**
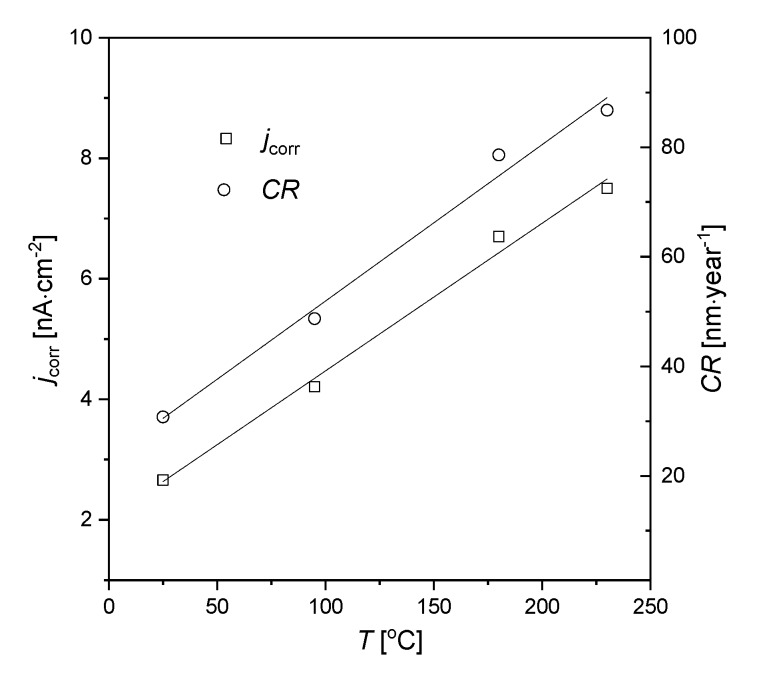
Corrosion current density (*j*_corr_) and corrosion rate (*CR*) for the AISI 1010 steel electrodes immersed in sulfolane at 25, 95, 180, and 230 °C.

Corrosion potential is a thermodynamic value that provides the information about the corrosion tendency of the material in a specific environment, while corrosion current density (*j*_corr_) is a kinetic value which is proportional to the corrosion rate (*CR*) (see Equation (5)). It was found that for the AISI 1010 steel electrodes immersed in sulfolane corrosion potential (*E*_corr_) corresponds to the open circuit potential. It was also stated that for a sulfolane temperature equal to 230 °C, the value of corrosion current density for tested steel is only ca. 7.5 nA·cm^−2^, which corresponds to a corrosion rate of 87 nm·year^−1^. However, an increase in the sulfolane temperature from 25 to 230 °C causes ca. 2.8-fold higher corrosion rate. Parameters *j*_corr_ and *CR* as a function of sulfolane temperature (*T*) are shown in Figure 3. One can see that both *j*_corr_ = *f*(*T*) and *CR* = *f*(*T*) dependencies are linear with the slopes of 0.29(2) nm·year^−1^/°C and 0.025(1) nA·cm^−2^/°C, respectively. The calculated slopes can be interpreted as the rate of change of *j*_corr_ and *CR* per degree Celsius. The obtained values of *E*_corr_, *j*_corr_ and *CR* indicate that the tendency to corrosion and corrosion rate of AISI 1010 steel increase with increased sulfolane temperature.

Generally, the decomposition temperature of sulfolane is ca. 230 °C. However, slow thermal decomposition with the formation of sulphur dioxide and butadiene already occurs at temperatures above 200 °C. Surprisingly, high decomposition rates may occur at temperatures of 175–190 °C for sulfolane contaminated with oxygen [24,31]. In the absence of water, sulphur dioxide does not react strongly with carbon steel however, in the presence of water and oxygen it forms highly corrosive sulfurous acid according to the following formula:

(8) $$C_{4}H_{8}O_{2}S+O_{2}+H_{2}O\to H_{2}{\mathrm{SO}}_{3}+C_{3}H_{7}{\mathrm{CO}}_{2}H$$

The calculated *β*_a_ and *β*_c_ values (see Table 2) indicate that for all investigated electrodes the rate determining step of the corrosion is the anodic process. For AISI 1010 steel electrodes anodic process can be associated with the iron oxidation:

(9) $$\mathrm{Fe}-2e^{-}\to {\mathrm{Fe}}^{2+}$$

Taking into account Equation (8), the cathodic process coupled with the anodic may be the reduction of H^+^ ions:

(10) $$2H^{+}+2e^{-}\to H_{2}$$

Using Equations (3) and (4) the Stern–Geary coefficients (*B*) and polarization resistance (*R*_p_) were calculated and are shown in Table 2. One can see that for the AISI 1010 steel electrode immersed in sulfolane at 25 °C, the *B* value is equal to 25 mV, which corresponds to the active corrosion state. However, for electrodes immersed in sulfolane at 95, 180 and 230 °C, relatively high values of the parameter *B* (70–130 mV) were obtained, indicating the passive corrosion state for those materials. This fact can be explained by the reaction of carbon steel with sulphurous acid during the corrosion process. It causes the formation of ferrous sulfite (FeSO_3_), which adheres to the steel surface and forms a layer that protects the alloy against further corrosion attack. Note that all obtained *R*_p_ values are of the order of MΩ·cm^2^. However, the highest polarization resistance was determined for the electrode immersed at 95 °C. The obtained results indicate that the protective layer formed on the surface of the AISI 1010 steel may be a physical barrier between the corrosive environment and the material improving the corrosion resistance of the latter. Nevertheless, an increase in the sulfolane temperature to 180 °C and next to 230 °C cause breakdown of the protective layer and increase in the corrosion degree.

### 3.2. Surface Analysis of AISI 1010 Steel 

**Figure 4:**
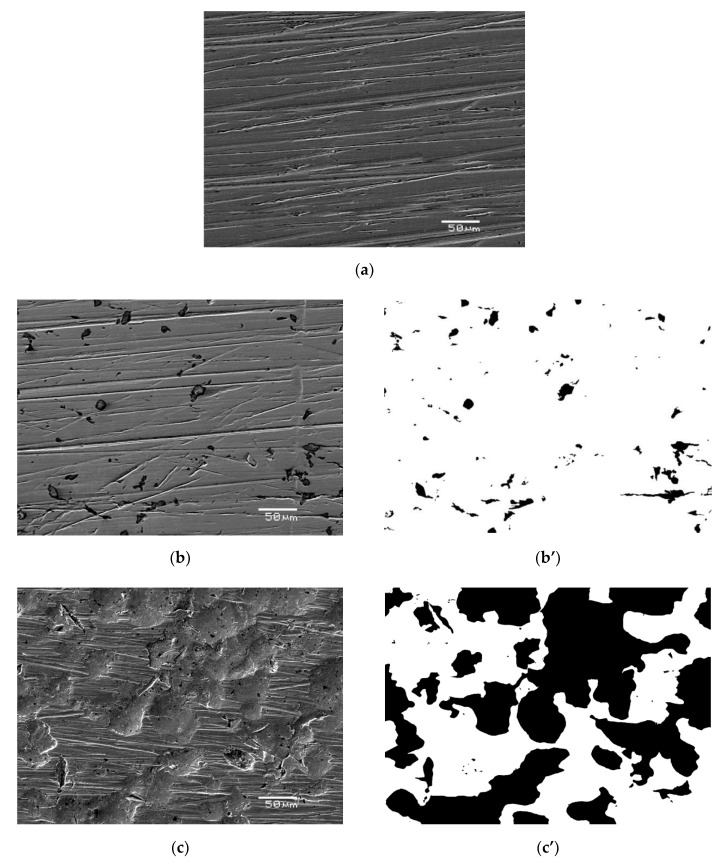
Exemplary scanning electron microscope (SEM) images for the AISI 1010 steel electrode in initial state (**a**) and immersed in sulfolane at 95 °C [25] (**b**) and 230 °C (**c**) as well as corresponding black and white images (**b’**, **c’**).

**Figure 5:**
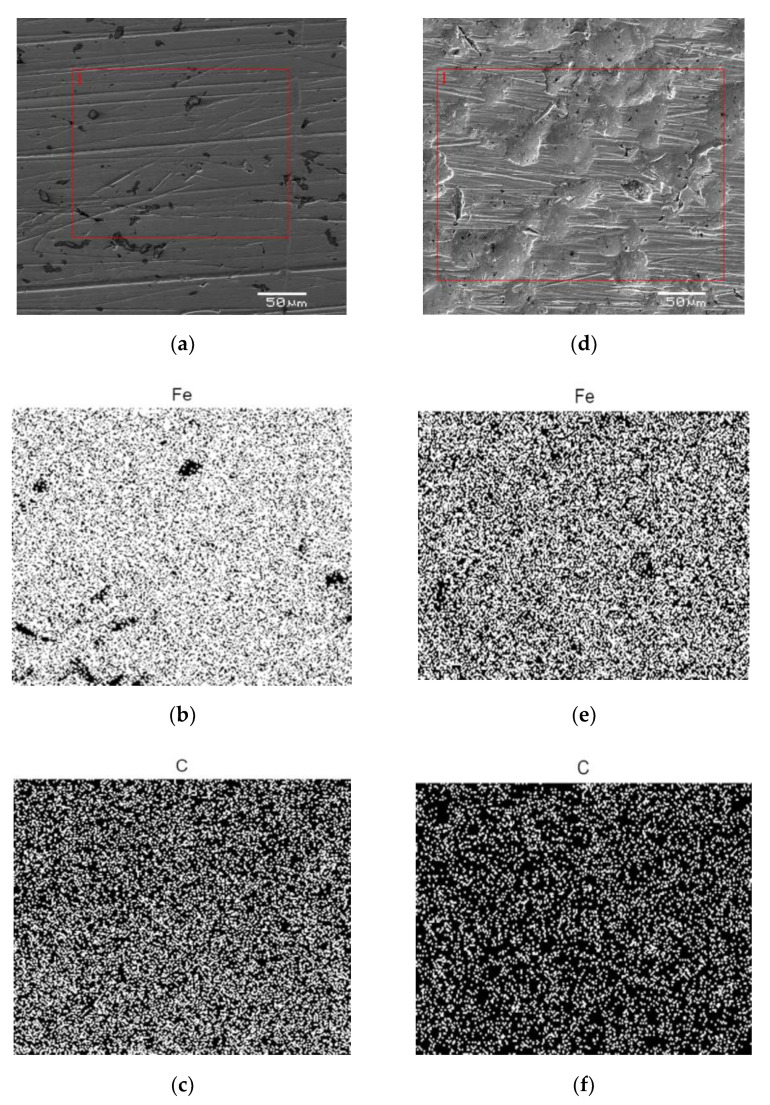
Exemplary images of iron and carbon elements distribution on the surface of AISI 1010 steel electrodes immersed in sulfolane at 95 °C (**a**–**c**) and 230 °C (**d**–**f**).

**Figure 6:**
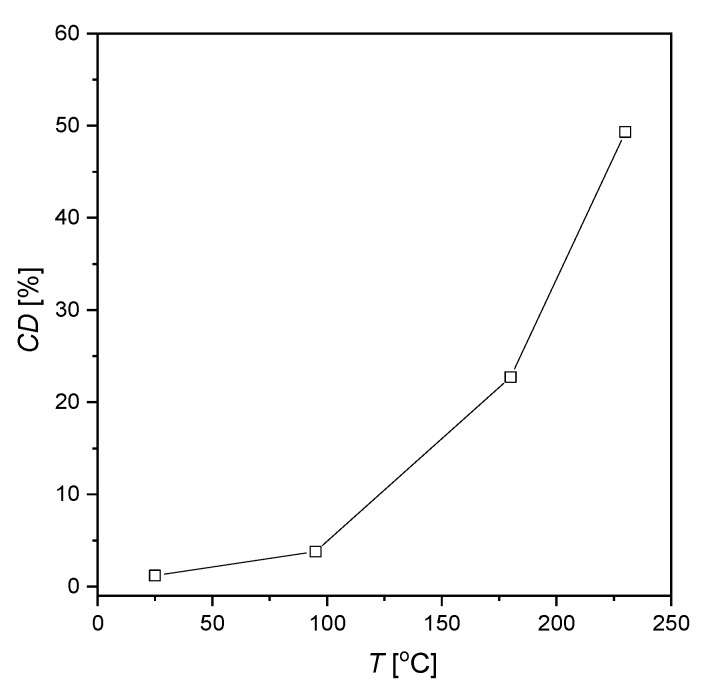
Corrosion degree (*CD*) for the AISI 1010 steel electrodes immersed in sulfolane at 25, 95, 180, and 230 °C.

The SEM/EDS images of the AISI 1010 steel surface without etching of corrosion products are shown in Figure 4 and Figure 5, respectively. For all investigated electrodes one can see numerous defects of different size and depth. Corrosion degree (*CD*) of AISI 1010 steel electrodes was estimated using SEM images and corresponding black and white images shown in Figure 4. As shown in Figure 6, the higher the temperature, the higher the corrosion degree. It was found that the corrosion degree of AISI 1010 steel immersed in sulfolane doubles approximately every 42 °C. Temperature increase leads to a higher corrosion rate (and hence higher corrosion degree) because cathodic and anodic reactions occur faster (see the *β*_a_ and *β*_c_ values in Table 2). Figure 5 shows distribution maps of iron and carbon elements on the AISI 1010 steel surface (without etching of corrosion products). The maps show that iron and carbon elements are homogeneously distributed on the material surface. However, the areas affected by corrosion are characterized by lower local amount of iron, which is obvious considering that it dissolves in such areas (see Equation (9)).

**Figure 7:**
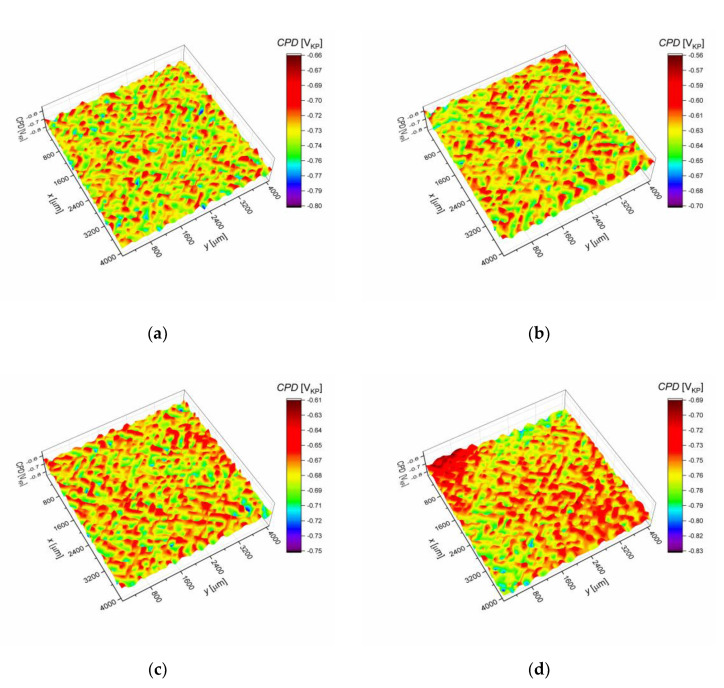
Contact potential difference (*CPD*) maps for the AISI 1010 steel electrodes immersed in sulfolane at 25 (**a**), 95 (**b**), 180 (**c**), and 230 °C (**d**); V_KP_ is the voltage measured versus Kelvin probe.

**Figure 8:**
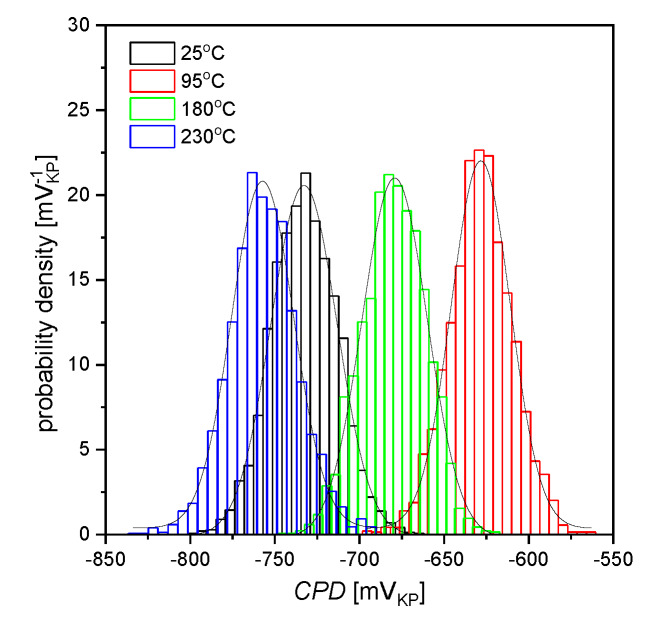
Histograms of contact potential difference (*CPD*) for maps shown in Figure 7; solid lines—fit of the Gaussian distribution; V_KP_ is the voltage measured versus Kelvin probe.

Contact potential difference (*CPD*) maps for the AISI 1010 steel electrodes are shown in Figure 7a–d. For each map, a corresponding histogram was prepared, as illustrated in Figure 8. Fitting the histograms with the Gaussian function (Equation (7)) allowed to quantify the material surface properties. In particular, it enabled to determine the average value of contact potential difference (*CPD*_av_) and its standard deviation *σ* that are reported in Table 3.

The AISI 1010 steel electrode immersed in sulfolane at 25 °C (Figure 7a) is characterized by about 100 mV_KP_ lower *CPD*_av_ in comparison with the sample immersed in sulfolane at 95 °C (Figure 7b). Note that the sample immersed in sulfolane at 95 °C has the highest *CPD*_av_ value among all the investigated electrodes. This can be explained by the fact that a certain layer of corrosion product is formed on the electrode surface. However, further increase in the temperature (to 180 and 230 °C) results in the discontinuities of the corrosion product layer and a decrease in *CPD*_av_ value (see Figure 7c,d). AISI 1010 steel electrode immersed in sulfolane at 230 °C (Figure 7d) is characterized by the smallest value of *CPD*_av_. Moreover, *CPD*_av_ and *R*_p_ as a function of temperature are characterized by a similar trend. It was also found that the spread of the *CPD* distribution from the average (represented by *σ*, Table 3) is the smallest for the AISI 1010 steel electrode immersed in sulfolane at 95 °C and increases with increased temperature. The obtained results show that the AISI 1010 steel electrode immersed in sulfolane at 95 °C is characterized by the most uniform surface among all the investigated electrodes.

## 4. Conclusions

1. Thermodynamic tendency for the corrosion process, as well as the corrosion rate of the AISI 1010 steel, increase with increased sulfolane temperature. In particular, an increase in the sulfolane temperature from 25 to 230 °C results in a decrease in corrosion potential value by ca. 175 mV (Figure 1, Table 1) and, simultaneously, a 2.8-fold increase in the corrosion rate (Figure 3, Table 2).
2. The highest polarization resistance (Table 2) and average contact potential difference (Table 3) were found for the AISI 1010 steel immersed in sulfolane at 95 °C; thus this electrode is characterized by the most compact and uniform surface among all the investigated ones.
3. The corrosion product layer formed on the surface of the AISI 1010 steel electrodes in sulfolane is a physical barrier between the corrosive environment and the material and partially protects the alloy against further corrosion attack.
4. An increase in the sulfolane temperature caused a gradual breakdown of the protective layer and an increase in the corrosion degree. It was found that the corrosion degree of AISI 1010 steel immersed in sulfolane doubles approximately every 42 °C (Figure 6).

## Figures and Tables

**Table 1:** Average value of open circuit potential (*OCP*_av_) and corresponding standard error (*SE)* determined for the AISI 1010 steel electrodes immersed in sulfolane at 25, 95, 180, and 230 °C; V_SCE_ is the electrode potential measured versus saturated calomel electrode.

Parameter	25 °C	95 °C	180 °C	230 °C
*OCP*_av_ (mV_SCE_)	−81.2	−103.8	−152.6	−255.7
*SE* (mV_SCE_)	0.7	0.5	0.3	1.2

**Table 2:** Electrochemical parameters for the AISI 1010 steel electrodes immersed in sulfolane at 25, 95, 180, and 230 °C; V_SCE_ is the electrode potential measured versus saturated calomel electrode.

Temperature (°C)	*E*_corr_ (mV_SCE_)	*j*_corr_ (nA·cm^−2^)	*β*_a_ (mV_SCE_)	*β*_c_ (mV_SCE_)	*B* (mV_SCE_)	*R*_p_ (MΩ·cm^2^)
25	−85.6(4)	2.6(1)	219(14)	80(2)	25	9.6
95	−104.1(4)	4.2(2)	776(46)	485(22)	130	30.8
180	−154.5(2)	6.8(2)	471(14)	299(6)	79	11.7
230	−242.1(7)	7.5(1)	336(52)	318(43)	71	9.5

**Table 3:** Statistical parameters for the contact potential difference (*CPD*) maps of the AISI 1010 steel electrodes immersed in sulfolane at 25, 95, 180, and 230 °C; *CPD*_av_ is the average value and *σ* is the standard deviation; V_KP_ is the voltage measured versus Kelvin probe.

Parameter	25 °C	95 °C	180 °C	230 °C
*CPD*_av_ (mV_KP_)	−732.8(4)	−628.3(4)	−679.2(4)	−757.3(3)
*σ* (mV_KP_)	19.6(5)	17.5(5)	18.4(5)	19.3(6)
